# Tensile Strength and Degradation of GFRP Bars under Combined Effects of Mechanical Load and Alkaline Solution

**DOI:** 10.3390/ma13163533

**Published:** 2020-08-11

**Authors:** Qingping Jin, Peixia Chen, Yonghong Gao, Aihua Du, Dongxu Liu, Lizhi Sun

**Affiliations:** 1 School of Urban Construction, Wuhan University of Science and Technology, Wuhan 430065, China; jinqingping@wust.edu.cn (Q.J.); chenpeixia@httzjt.com (P.C.); gaoyonghong1967@163.com (Y.G.); 2 School of Civil Engineering, Shandong Jianzhu University, Jinan 250101, China; 3 Department of Civil and Environmental Engineering, University of California, Irvine, CA 92697, USA; dongxul@uci.edu (D.L.); lsun@uci.edu (L.S.)

**Keywords:** GFRP bars, corrosion, damage, mechanical properties, degradation

## Abstract

Mechanical properties of glass fiber reinforced polymer (GFRP) composites degrade under the combined effects of mechanical load and alkaline solution, affecting the service ability and safety of GFRP reinforced structures. In this study, GFRP bars were loaded with cyclic tension at different stress levels and immersed in alkaline solution for days to investigate the tensile properties and degradation law of GFRP bars. The degradation mechanisms were studied at micro-, meso- and macro-scales with scanning electron microscopy (SEM) and three-dimensional X-ray microscopy, respectively. The results show that tensile strength and degradation rate of GFRP bars are mainly dependent on the different stress levels and alkaline solution. When stress level is higher, the tensile strength degrades more quickly, especially in the early stages of soaking. With the loading and immersion time, the elastic modulus and Poisson’s ratio increase at first and then decrease. The ultimate tensile strain is relatively stable, whereas the ultimate elongation is significantly reduced. A strength-degradation model was proposed and fit well with experimental data, demonstrating that the model can be applied to predict tensile strength degradation under combined effects of the load and alkaline solution.

## 1. Introduction

Glass fiber-reinforced polymer (GFRP), composed of resin and glass fibers, has many advantages such as light weight, high strength and diamagnetism [1], which can be applied as alternative or supplementary material for steel rebar in civil engineering [2]. However, mechanical properties degradation can affect the overall durability of GFRP bars [3]. GFRP structures are often subjected to various harsh environments (e.g., acid rain, alkaline environment in concrete and seawater) in their service; therefore, its performance might be degraded to certain extent, adversely structural performance and safety are affected. 

The environmental effects on GFRP’s performance [4,5] were studied in the last decades, including changes of temperature and humidity [6,7] and chemical corrosion from acidic, alkaline and/or salt [8,9]. While the individual degradation performance and corresponding models of GFRP bars were explored, there were differences in test conditions and methods, resulting in inconsistent conclusions on the underlying mechanisms. Through alkali-, acid- and salt-resistance tests, the result was found that tensile strength degradation of GFRP bars is more severe in alkaline environment. The degradation degree was related to corrosion time [10,11]. The effect of alkaline environment on mechanical properties of GFRP bars at different temperatures conditions was also studied; the result show that ultimate tensile strength decreased significantly with the temperature increasing at the same immersion time. As immersion time increased in alkaline solution, the strength degraded more, and the effect of corrosion time on the strength was greater than that of temperature [12,13,14,15].

GFRP reinforced structures are frequently subjected to the combined effect of the load and corrosive environment in their service. When loading stress reaches a certain value, the fibers inside GFRP bars can break, escalating the effect of corrosive environment that destroys the interface between the resin and fibers. The load causes plastic deformation in the matrix, which tends to lower the rate of fiber-breakage evolution in the form of energy dissipation [16,17]. To test the effect of the environment and stress on the mechanical properties of GFRP bars, Nkurunziza et al. [18] put GFRP bars in distilled water in an accelerated experiment with a certain stress level. The strength of GFRP bars did not decrease significantly under humid environment and stress. However, Sen et al. [19] exposed GFRP bars to an alkaline solution and sustained 10% stress level on specimens and observed the significant degradation of tensile strength of GFRP bars during the test. Xue et al. [20] also found that, when GFRP bars were subjected to different stress levels in an alkaline environment, the alkaline solution caused internal compactness rate of GFRP bars to decrease, and, with the increase of stress level, the reduction was more obvious. The above research demonstrates that the combined effects of alkaline solution and the loading on the tensile properties of GFRP bars are not much different from the single factor effect of alkaline environment within a certain period of time and at a specific stress level. When the stress exceeds a certain level, the corrosion resistance of the bars is obviously degraded. Internal damage develops with a threshold of stress, and residual strain evolves gradually along with internal damage development [21]. Under the combined effects of environment and sustaining stress, the failure mechanisms of GFRP can be divided into medium diffusion mode under low stress condition, matrix-cracking mode with intermediate stress level and stress control mode under high stress. When sustaining stress is small, the resin may not crack, and the erosive medium can only enter inside the bars by the diffusion. When stress level is high, the resin matrix formed micro-cracks, corrosion resistance of GFRP bars is mainly affected by the stress and the service life of GFRP bars is greatly reduced [22]. 

Generally, the mechanical performance of GFRP bars is greatly affected by the temperature and stress levels in alkaline environment. Most studies focus on mechanical properties by accelerated test and pay little attention to the combined effects between the load and alkaline solution in natural environment. In this study, some short-term loads were considered: GFRP bars were loaded and immersed in alkaline solution to study mechanical properties and establish life prediction of GFRP bars under combined effects of the mechanical load and alkaline environment. During the whole experiment, the temperatures were consistent with natural environment temperatures. Microscopic imaging techniques were used to characterize the internal microstructure of GFRP bars. Ultimate tensile strength was tested, and degradation mechanisms of tensile strength were obtained under the combined effects of the load and alkaline solution. Based on the Arrhenius equation, a modified life prediction model was proposed and validated for the GFRP ultimate tensile strength. 

## 2. Experimental Method

### 2.1. Materials

**Figure 1:**
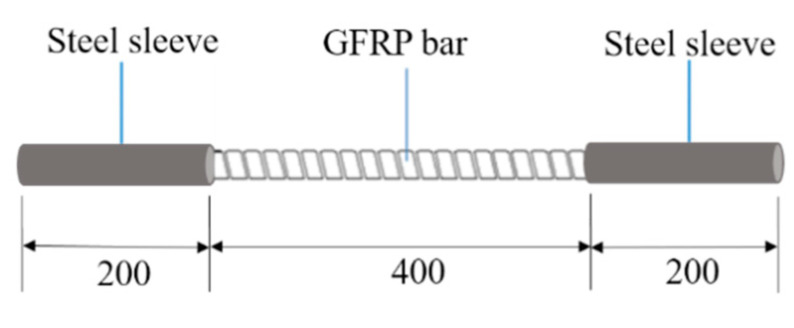
Test specimen [mm].

Experimental materials were 16 mm-diameter GFRP bars with thread on the surface (Figure 1). The supplier was Shenzhen Oceanpower New Material Technology Co., Ltd (China). The volume fraction of fibers was 68.6%. The glass fiber was E-glass. GFRP was vinyl resin reinforced with 20–30-μm-diameter glass fibers. Full length of test specimen was 800 mm. They were steel sleeved with the length of 200 mm (L_1_) and the wall thickness of 3 mm at both ends of the bars. The effective test length was 400 mm (L_0_). 

### 2.2. Experiment Procedures

The alkaline solution was prepared according to Procedure A in ACI 440.3R-04 [23], and pH was approximately 12.7. The components of alkaline solution are shown in Table 1. Corrosion protection tube was set to protect the sleeve from the corrosion of alkaline solution during the experiment. 

GFRP bars were divided into four groups and subjected to cyclic tensile loading of 0%, 20%, 40% and 60% of ultimate failure load ($F_{b}$). A predetermined force was loaded onto some group of bars and then unloaded to zero. The loading and unloading procedures were repeated five times. Then, the bars were treated with an anti-corrosion treatment and immersed in alkaline solution. The immersion was from October to April in the natural environment. The temperature had no obvious differences during this period. The temperature range was as follows: the lowest average temperature was 2 °C, the highest average temperature was 33 °C and the average temperature was 15.4 °C. After GFRP bars were immersed in alkaline solution for 30, 90 and 180 days, they were removed and tested. Tensile test on GFRP bars was carried out according to ACI 440.3R-04 [23]. Displacement-controlled loading mode was used with displacement stretching rate of 2 mm/min. The test was conducted by displacement control. Strain gauges were pasted at 1/2 and 1/4 of the effective test length of the specimen to test the strain.

## 3. Results and Discussion

### 3.1. Initial Structure of GFRP Bars

**Figure 2:**
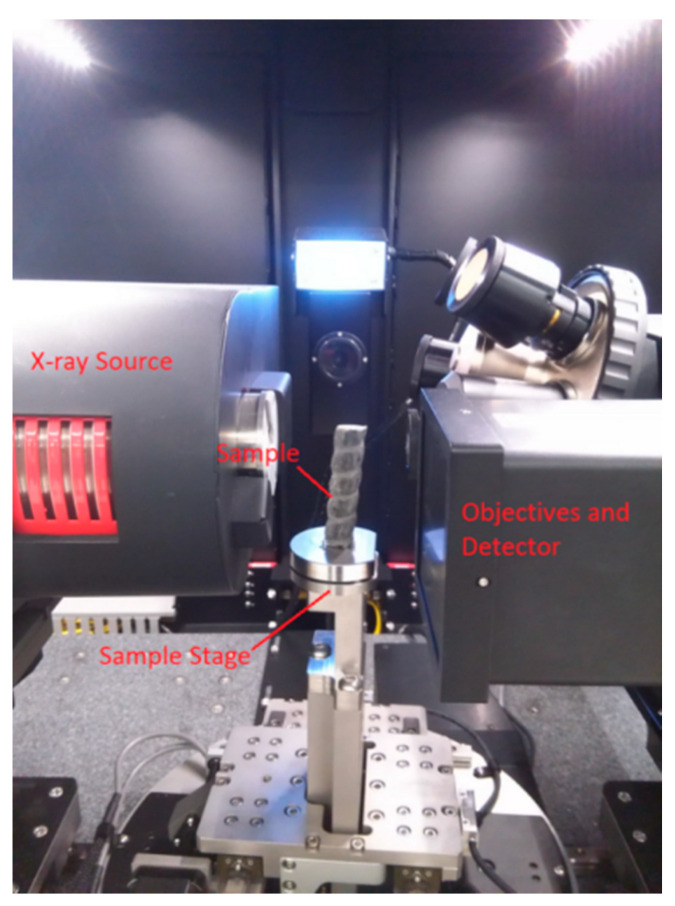
Zeiss 410 Versa XRM and the sample fixed in position.

**Figure 3:**
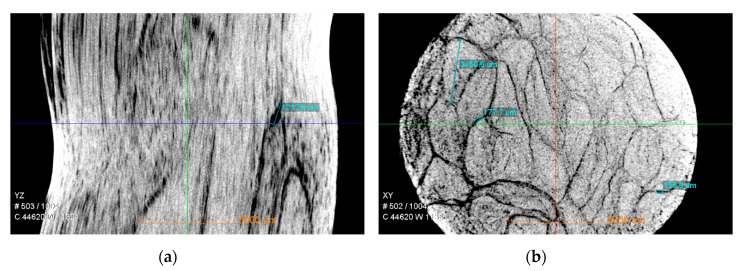

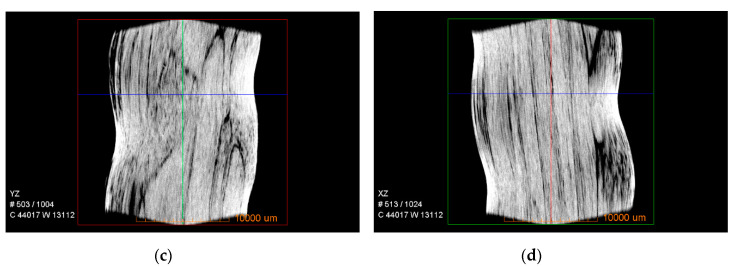
3D CT images of the internal structure of GFRP bars of: (**a**) YZ-plane; (**b**) XY-plane; (**c**) YZ-plane; and (**d**) XZ-plane.

Three-dimensional (3D) X-ray microscopy (Figure 2, Versa 410 Nano-CT scanner, Carl Zeiss X-ray Microscopy, Inc.) was used to image GFRP bars with the voxel size of 25 µm before the corrosion. The Nano-CT scanner can analyze the structure from 3D angles by tomography, and the size can reach sub-micrometers. It can clearly reflect the internal structure of the bar. The 16-mm-diameter bar was placed on the sample stage in the Nano-CT, as shown in Figure 2. With the sample rotation step of 0.225°, 1600 projections were acquired to reconstruct the 3D CT images. The appropriate resolution was set to obtain the internal crack distribution and micro-structure of the GFRP bar, as shown in Figure 3. YZ-plane indicates the direction perpendicular to the X-ray direction from the side, XZ-plane along the X-ray direction and XY-plane direction perpendicular to the X-ray direction from the top.

Figure 3 shows that there were many cracks in the original GFRP bars, and there were many defects in the production. The cracks were distributed over the entire section, but the cracks were obviously larger near the outer edge of the bars. In the central area, it can be seen from the three-dimensional illustration that the cracks were relatively small and thin. Overall, the initial crack defects resulted in uneven cross-section of the entire bar, loose structure at the edge and relatively dense central core area. 

### 3.2. Apparent Phenomena of GFRP Bars after the Corrosion 

After the bars were corroded in the alkaline solution for 15 days, it was obviously observed by naked eye that there was a small amount of white attachment on the surface of the bars, mainly because the resin and interface layer on the surface of the bars reacted with OH^−^ in alkaline solution, and a small amount of calcification was precipitated on the surface of the bar. After the combined effects of the load and alkaline solution for 180 days, a large amount of white precipitates appeared on the surface of the bars. The bars produced micro-cracks under the load damage. During the immersion process, the ions reacted inside the bars through the micro-cracks. OH^−^ in alkaline solution reacted with the glass fibers to form a silicate gel, and more calcification was also precipitated on the surface of the bars. It was found that the surface of the bars became extremely soft, the fibers and the resin were severely separated and pitting phenomenon on the surfaces was obvious.

### 3.3. Corrosion Reaction of GFRP Bars in Alkaline Solution

The degradation of mechanical properties of GFRP bar in alkaline solution were the result of the combination of water erosion and high concentrations of OH^−^. When water molecules penetrated the resin, the interfacial bond between the fibers and the resin were destroyed, the void of the resin was occupied and free volume of the composite was changed, which caused large cracks and hydrolyzed in the resin. The reaction equation is shown as Equation (1):

(1) $${R-\mathrm{COO}-R\prime }^{\;}+H-\mathrm{OH}\to {R-\mathrm{COOH}+R\prime }^{\;}-\mathrm{OH}$$

Although the resin had the protective function for the glass fibers, there were still water molecules and ${\mathrm{OH}}^{-}$ penetrating through the resin to the fiber surface, which caused damage to the ${\mathrm{SiO}}_{2}$ network skeleton. The chemical reaction equations are shown as Equations (2) and (3):

(2) $${\mathrm{SiO}}_{2}{+2H}_{2}O\to H_{4}{\mathrm{SiO}}_{4}$$

(3) $${2\mathrm{OH}}^{-}{+\mathrm{SiO}}_{2}\to {\mathrm{SiO}}_{3}^{2-}{+H}_{2}O$$

As the corrosion time increased, the interface between the fibers and resin in the alkaline solution was destroyed, the debonding phenomenon became more serious and the structural tightness decreased. In addition, water molecules or ${\mathrm{OH}}^{-}$ continually reacted with internal fibers to destroy the silica network skeleton and reduce the strength of the bars. In alkaline solution, many ${\mathrm{OH}}^{-}$ were contained, and the silicon–oxygen bond could chemically react with ${\mathrm{OH}}^{-}$, causing the destruction to of the silicon–oxygen and silane coupling agent (Equation (4)):

(4) $${\mathrm{Si}-O-\mathrm{Si}+\mathrm{OH}}^{-}\to {\mathrm{Si}-O}^{-}+\mathrm{Si}-\mathrm{OH}$$

The resin easily hydrolyzed in alkaline solution (Equation (5)):

(5) $${\mathrm{RCOOR}\prime }^{\;}+\mathrm{NaOH}\to {\mathrm{RCOONa}+R\prime }^{\;}\mathrm{OH}$$

Therefore, the interface between the glass fiber and resin and was subjected to varying degrees of corrosion damage under alkaline conditions, resulting in a decrease in the overall mechanical properties of the reinforcement. 

When the bars were immersed in alkaline solution, the corrosive liquid did not gradually diffuse into the bars from the outer surface. For the initial cracks inside the bars (Figure 3), the penetration speed was faster along the cracks. On the one hand, when Fick’s law was used to investigate the performance changes of the bars in alkaline solution, the permeability was not the true penetration rate of the corrosive liquid in the bars. The corrosion of the bars was based on the existing cracks from all directions simultaneously, not unidirectional osmosis corrosion. In the central core region of the bars, the crack was relatively small, and the diffusion velocity of alkaline solution was slow relative to the outer part. Under the periodic loading, when tensile load was low, no new cracks were generated in the bars. When tensile load was in a certain range, the loading only caused the original crack to open and close repeatedly. At this time, the main corrosion of the bars was still the part where the initial cracks exist, and, therefore, the low load did not significantly increase the corrosion rate of the bar. When tensile load reached a certain value, it caused the sizes of the cracks to enlarge in the length and width directions, which provided a channel for solution penetration and made it easier for the solution to enter the inner part of the bar, thereby aggravating the corrosion effect. Under high tensile load, corrosion resistance of GFRP bars was further deteriorated.

### 3.4. Mechanical Properties of GFRP Bars under Loading and Alkaline Solution

**Figure 4:**
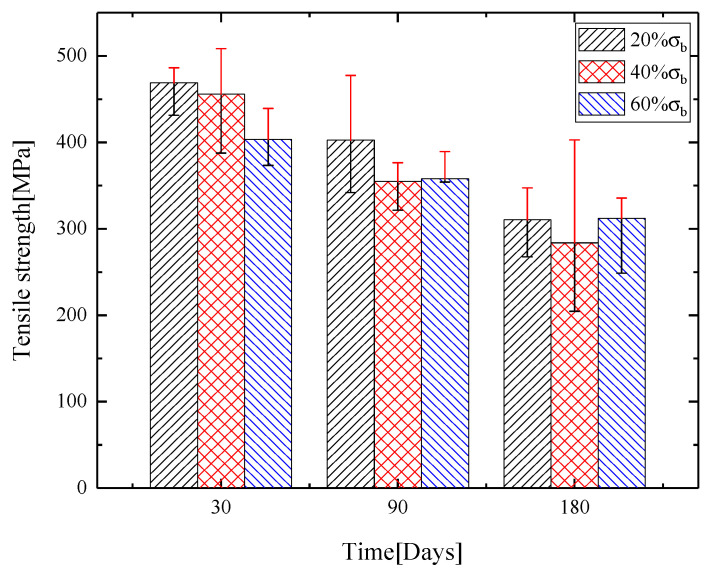
Tensile strength and standard deviation of GFRP bars.

GFRP bars were damaged by tension load; immersed in alkaline solution for 30, 90 and 180 days; and tested until the failure. The bars without the damage from the load were immersed for 180 days. The results are shown in Table 2. For GFRP bars, the volume fraction of fibers was 68.0%, tensile strength was 554.0 MPa and elastic modulus was 33.9 GPa. Under the 60% $\sigma_{b}$ (ultimate tensile strength) cyclic loading, GFRP bars immersed for 180 days became brittle. At this time, tensile strength was about 312.24 MPa, i.e. a strength retention ratio of 56.4%. The bars under 60% $\sigma_{b}$ loading are not listed in Table 3. Tensile strength and standard deviation of GFRP bars after the immersion in alkaline solution are shown in Figure 4.

**Figure 5:**
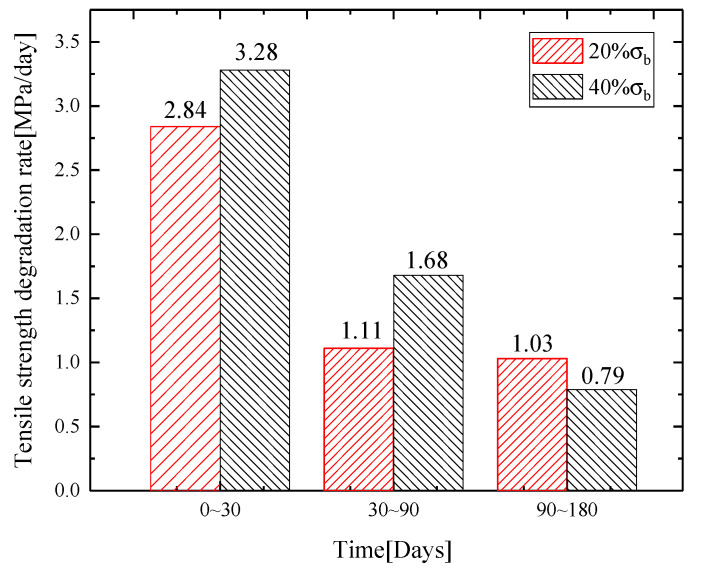
Strength degradation rate of GFRP bars.

Table 2 and Figure 4 show that, as immersion time in the alkaline solution increased, ultimate tensile strength of the bars gradually decreased. GFRP bars were immersed for 30 days. Through the tensile test, the ultimate strength and strain of reinforcement were obtained. The results show that tensile strength degradation increased with the increase of cyclic stress level. Under the cyclic loading of 20% and 40%, ultimate tensile strength of the bar was reduced, respectively, by 15.4% and 17.8% compared with the one-time tensile experiment after immersion for 30 days. After 90 days, ultimate tensile strength of the bars decreased by 27.4% and 36.0%. After 180 days, ultimate tensile strength of the bars decreased by 44.0%% and 48.8%. The strength degradation rate is shown in Figure 5. During the experiment period of 0–30 days, strength degradation rates of the bars were 2.84 and 3.28 MPa/day, respectively, under the cyclic loading of 20% $\sigma_{b}$ and 40% $\sigma_{b}$. During 30–90 days, strength degradation rates of the bars were 1.11 and 1.68 MPa/day. During 90–180 days, strength degradation rates of the bars were 1.03 and 0.79 MPa/day. The analysis showed that, with the two levels of stress cyclic loading, combined with alkaline solution, tensile strength decreased significantly during the period of 0–90 days, and then the changes slowed down after 90 days. The degradation was fastest during the period of 0–30 days because the firm molecular structure and chain structure were more difficult to break with the increase of reaction time. Under cyclic loading, the bars were damaged, and micro-pores and micro-cracks were formed. After long-term cyclic loading, the macroscopic expression was damage accumulation, i.e. the micro-voids and micro-cracks expanded, decreasing the durability of the material. At the same time, the inside of the bars also generated many micro-cracks and expanded, accompanied by resin cracking, fiber breakage and fibers detaching from the resin. With the loading, alkaline solution was diffused into the bars through micro-pores and micro-cracks, which accelerated the interface corrosion, and the bonds between the resins and the fibers were weakened. As the cyclic loading increased, the damage degree of the bars also increased, eventually resulting in a decrease of the tensile strength. After 40% $\sigma_{b}$ cyclic loading, ultimate tensile strength was greatly reduced by immersing in alkaline solution, which indicated that increasing the cyclic load can increase the damage to the fibers and resins, accelerate the hydrolysis reaction of the resins and alkaline solution and reduce the bonding force between the fiber and resin, resulting in the tensile strength degradation of GFRP bars.

Elastic modulus and Poisson’s ratio showed the discreteness. After 30 days, the elastic modulus of the bars increased obviously with the increase of cyclic loading. After the coupling of 90 days, elastic modulus of the bars showed a decreasing trend with the increase of the cyclic loading. Under the same damage loading, with the increase of coupling time, both elastic modulus and Poisson’s ratio increased at first and then decreased. As the loading damage degree increased, the Poisson’s ratio of the bars decreased slightly at the same time. It indicated that the damage of the bars was caused by the internal interface damage, fracture of glass fibers, longitudinal deformation and lateral deformation of the resin. The lateral deformation ability was obviously reduced. 

Table 2 shows that the corrosion time had little effect on the elongation of the bars, while the elongation change under different cyclic loading at the same corrosion time was relatively small, which indicated that, under the combined action of cyclic loading and alkaline solution, the damage of the cyclic loading on the glass fiber was greater than that on the resin.

Ultimate tensile strain of 20% $\sigma_{b}$, 40% $\sigma_{b}$ and 60% $\sigma_{b}$ showed significant decreasing trend after cyclic loading on GFRP bars in alkaline solution for 30 days. At this time, the influence of cyclic loading on the bars was the main factor. After 90 days, ultimate tensile strain of the bars tended to be stable.

## 4. Degradation Model of Tensile Strength of GFRP Bars

**Figure 6:**
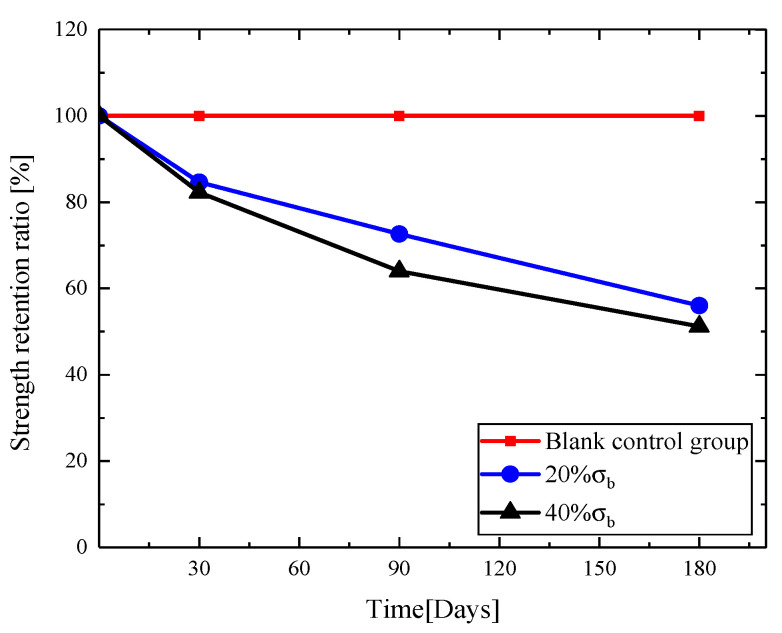
Tensile strength retention ratio of GFRP bars under different loadings.

According to the experiment data, the curves of tensile strength retention ratio with time under the cyclic loading of 20% $\sigma_{b}$ and 40% $\sigma_{b}$ were obtained, as shown in Figure 6. 

Based on the short-term experiment data, to predict the durability of GFRP bars in the long-term service, Arrhenius equation [24] was used to establish the prediction model (Equation (6)).

(6) $$k=\frac{df}{dt}=A_{0}\exp(\frac{-E_{a}}{RT})$$

where *k* is the reaction rate at the corrosion reaction temperature *T*, *t* is corrosion time, *f* is the tensile strength of GFRP bars at time *t*, *A*_0_ is a constant related to material properties and degradation process, *E*_a_ is the activation energy causing degradation of tensile strength of GFRP bars, *R* is molar gas constant and *T* is the absolute temperature of the environment. 

Taking logarithms on both sides of Equation (6) yields:

(7) $$\ln(\frac{1}{k})=\frac{-E_{a}}{RT}-\ln A_{0}$$

Equation (7) shows that the time logarithm is linear with the reciprocal of temperature, with the slope of $\frac{E_{a}}{R}$. Let the tensile strength of GFRP bars at $t_{0}$ be $f_{0}$ and at $t_{1}$ be $f_{1}$; integrating Equation (6) obtains Equation (8):

(8) $$f_{1}-f_{0}=A_{0}\exp(\frac{-E_{a}}{RT})\cdot t$$

Equation (9) can be obtained by taking logarithms simultaneously on both sides of Equation (8).

(9) $$\mathrm{lg}t=\mathrm{lg}\frac{f_{1}-f_{0}}{A_{0}}+(\frac{E_{a}}{R}\cdot \mathrm{lg}e)/T$$

The relationship between the strength retention ratio of the GFRP bar (the percentage of the residual tensile strength of the bar relative to the initial tensile strength) and the duration of the accelerated experiment is defined by exponential function (Equation (10)).

(10) $$Y=100\exp(\frac{-t}{\tau })$$

where *Y* is the strength retention ratio of the bar, *t* is the corrosion time and τ is $\frac{1}{k}$. It is assumed that GFRP bar is completely destroyed when corrosion time is infinite.

**Figure 7:**
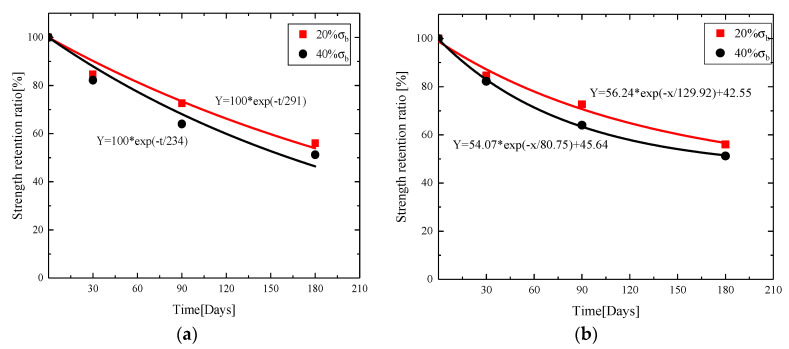
Fitting curve of strength retention ratio of GFRP bars in alkaline solution under different loading of: (**a**) classical model; and (**b**) modified model.

According to Equation (10), the nonlinear fitting of Figure 6 was carried out to obtain the curve of the retention rate of GFRP bars in alkaline solution under different loading conditions, as shown in Figure 7a. After varying degrees of cyclic loading damage, the initial tensile strength of the bars decreased significantly more than that of the later stage. Thus, the classical model is modified to obtain Equation (11), named the modified model, and the corrected fitting curve is shown in Figure 7b.

(11) $$Y=A_{1}\cdot \exp(\frac{-t}{\tau }){+y}_{0}$$

Comparing the classical model and the modified model in Figure 7a,b, the value of A is 100 in classical model, but it is varied in modified model. A_1_ is an equation coefficient that indicates the multiple effect of strength degradation rate and the time. τ (days/MPa) is the reciprocal of K, meaning the degradation time per MPa. The classical model is not applicable for the combined effects of the load and alkaline solution. By fitting data, the result shows that the parameters of modified model reduce and the curvature increases, which can better reflect the effect of load damage. The constant $y_{0}$ is used to adjust the location of the curve on the longitudinal axis, i.e., the strength retention ratio, which reflects the direct effect of the load on strength degradation.

Table 3 lists the corresponding fitting parameters and correlation coefficient squared ($R^{2}$) of the two models, and $R^{2}$ is close to 1. Table 3 shows that correlation coefficient of the degradation curve fitting under the classical model is about 0.95, while the correlation coefficient of modified model is above 0.99, i.e., the fitting curve is better than the classic model. According to the experiment results, the degradation rate of ultimate tensile strength of GFRP bars tended to decrease with the increase of corrosion time. The modified model curve decreases rapidly at the beginning and tends to slow down at the later stage. Table 4 also shows that, under the classical model and modified model, τ values corresponding to 40% $\sigma_{b}$ are less than 20% $\sigma_{b}$. $\tau =\frac{1}{k}$ and k are the reaction rate of GFRP bars exposed to the corrosion solution, and the reaction rate of the bars exposed to alkaline solution under 40% $\sigma_{b}$ loading damage is greater than 20% $\sigma_{b}$. Thus, greater damage to the bars leads to a faster reaction rate between the bars and corrosive solution and more serious deterioration of mechanical properties of the bars, which agrees with the results obtained from the experiment. 

**Figure 8:**
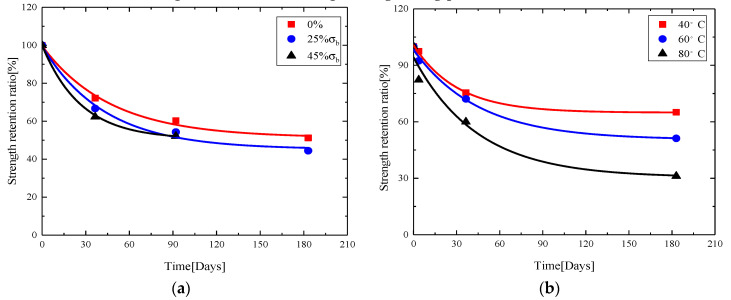
Fitting of modified model with experimental data of: (**a**) the results in [20]; and (**b**) the results in [25].

Based on the modified Arrhenius degradation model proposed in this paper, the tensile strength retention ratios of GFRP bar exposed to alkaline environment in [20,25] were fitted. The fitting results are shown in Figure 8. Figure 8a is a fitting curve for tensile strength retention ratio of GFRP after sustained loading for 183 days at different stress levels in alkaline solution at 60 °C. Figure 8b is a fitting curve for tensile strength retention ratio of GFRP bars after accelerated corrosion in alkaline solution at different temperatures. The corresponding fitting parameters are shown in Table 4.

Figure 8 and Table 4 show that the modified model fits well the tensile strength retention ratio of GFRP bars under different stress levels and different temperatures; the square of correlation coefficient is above 0.97. To verify the applicability of modified model proposed, the modified equation was fitted with the experimental data found in the literature, and the fitting curve could pass more experimental values. Table 5 lists the fitting coefficients. Except for a few cases, the overall fitting is better, and the squares of correlation coefficients are above 0.96, indicating that the modified degradation model is suitable for predicting tensile strength of GFRP bars under long-term corrosion. The parameters $A_{1}$, τ and $y_{0}$ need to be determined by a large number of experiments. 

## 5. Conclusions

In this study, mechanical properties of GFRP bars were tested under the combined effects of mechanical load and alkaline solution. A modified model for life prediction was proposed according to Arrhenius equation. The following conclusions can be drawn.

(1) SEM analysis showed that initial cracks of GFRP bars were larger in the outer edge and smaller in the inner edge. Consequently, the delamination and microcracks were generated and additional water and chemicals diffused through the defects. The increase in pores was attributable to the subsequent degradation of the fiber–matrix interface and surface corrosion.
(2) Under the combined effects of the load and alkaline solution, tensile strength of GFRP bars decreased more quickly during the experiment period of 0–30 days. During 30–90 days, intensity degradation rate decreased 50%, whereas it almost did not change from 90 to 180 days. The Ultimate tensile strain was relatively stable, but the ultimate elongation was significantly reduced.
(3) Tensile strength of GFRP bars degraded more quickly under combined effects of the different stress levels and alkaline solution. The greater stress level led to more serious damage and faster strength degradation of the bar. The elastic modulus and Poisson’s ratio of GFRP bars increased at first and then decreased with the loading and immersion time. The ultimate tensile strain was relatively stable, but the ultimate elongation was significantly reduced.
(4) A modified model was proposed according to the experimental results. The effects of alkaline solution and loading damage were expressed by parameters in this model. Moreover, the model indicated good agreement with the experiment results from other studies. Therefore, it can predict the tensile strength of GFRP bar exposed to alkaline solution under cyclic loading.

This study did not consider the durability of GFRP bar under the sustained stress state, which is common in some real engineering structures. These studies are of great significance to the real structural design calculation and performance evaluation. In the future, the research in this field should be strengthened to serve better the real structure construction.

## Figures and Tables

**Table 1:** The components of alkaline solution [g/L].

Ca(OH)_2_	NaOH	KOH
118.5	0.9	4.2

**Table 2:** Mechanical properties of GFRP bars under loading and in alkaline solution.

Corrosion Time [day]	Stress Level [%]	Average Tensile Strength [MPa]	Average Elastic Modulus [GPa]	Poisson’s Ratio	Elongation [%]	Ultimate Tensile Strain [%]	Strength Retention Ratio [%]
30	20	468.85	34.10	0.30	3.388	1.4	84.62
	40	455.66	37.54	0.27	3.444	1.2	82.24
	60	403.51	48.94	0.41	3.066	0.8	72.83
90	20	402.47	40.12	0.31	3.265	1.0	72.64
	40	354.70	39.14	0.32	3.354	0.9	64.02
	60	357.75	36.89	0.25	3.288	1.0	64.58
180	0	455.46	37.58	0.30	3.22	1.2	82.21
	20	310.34	34.54	0.28	2.918	1.1	56.02
	40	283.70	33.15	0.23	3.005	1.1	51.21
	60	-	—	—	—	—	-

**Table 3:** Strength fitting equation coefficient of GFRP bar for different models.

Cyclic Loading Level		20% $\sigma_{b}$	40% $\sigma_{b}$
Classical model	τ	291	234
	$R^{2}$	0.9650	0.9465
Modified model	$A_{1}$	56.24	54.07
	τ	129.92	80.75
	$y_{0}$	42.55	45.64
	$R^{2}$	0.9882	0.9992

**Table 4:** Fitting equation coefficient of modified model with experimental values in [20,25].

Literature		[20]			[25]		
		0	25% $\sigma_{b}$	45% $\sigma_{b}$	40 °C	60 °C	80 °C
Modified model	$A_{1}$	48.326	54.377	49.480	35.798	47.756	63.831
	τ	47.051	42.663	26.661	30.491	44.940	44.425
	$y_{0}$	51.302	45.218	50.520	64.863	50.468	30.417
	$R^{2}$	0.9941	0.9926	0.9995	0.9986	0.9945	0.9683

**Table 5:** Coefficient of modified model for fitting equation with experimental values of other studies.

Literature	Temperature (°C)	Stress (MPa)	Diameter (mm)	$A_{1}$	τ	$y_{0}$	$R^{2}$
[26]	30	0	sheet	64.444	73.046	35.012	0.9760
	40			43.994	25.442	54.621	0.9843
	50			44.888	13.933	53.457	0.9584
	60			47.844	10.518	50.044	0.9124
[27]	60	0	9.53	−3.228	−31.294	103.228	0.9996
[28]	60	0	12	35.886	47.268	63.729	0.9647
